# Acquired Amegakaryocytic Thrombocytopenia Presenting as Pancytopenia in a Patient With Chronic Lymphocytic Leukemia: A Case Report

**DOI:** 10.1155/crh/6735469

**Published:** 2026-09-18

**Authors:** Christopher C. Chen, Anand Shah, Marina Andrawis, Jared Benjamin, Manali Shah, Anthony Fratella-Calabrese, Victor T. Chang

**Affiliations:** ^1^ Department of Medicine, Rutgers New Jersey Medical School, Newark, New Jersey, USA, rutgers.edu; ^2^ Rutgers New Jersey Medical School, Newark, New Jersey, USA, rutgers.edu

**Keywords:** AAMT, acquired amegakaryocytic thrombocytopenia, case report, chronic lymphocytic leukemia

## Abstract

Acquired amegakaryocytic thrombocytopenia (AAMT) is a rare hematologic disorder characterized by severe thrombocytopenia due to an isolated absence of megakaryocytes in the bone marrow, with preservation of other hematopoietic lineages. We present an 84‐year‐old man with IgHV‐mutated chronic lymphocytic leukemia (CLL), diagnosed in 1997 and managed with observation until initiation of ibrutinib for treatment‐naïve CLL in 2024. He presented acutely with pancytopenia, for which a bone marrow biopsy showed marked absence of megakaryocytes. While platelet increments with transfusion were minimal and transient, additional treatments included dexamethasone, IVIG, cyclosporine, thrombopoietin receptor agonists, and reinitiation of ibrutinib. The patient eventually succumbed to gastrointestinal bleeding secondary to severe thrombocytopenia. This case underscores the diagnostic importance of early marrow evaluation in CLL patients with unexplained cytopenia. The pathogenesis of AAMT in CLL is likely multifactorial, involving immune dysregulation and impaired megakaryopoiesis. Management remains challenging due to lack of standardized guidelines and unpredictable treatment responses.

## 1. Introduction

Amegakaryocytic thrombocytopenia (AMT) is a rare hematologic disorder characterized by severe thrombocytopenia marked by isolated absence of megakaryocytes in the bone marrow, with relative preservation of other hematopoietic lineages [1]. AMT is often indistinguishable from immune thrombocytopenia (ITP), as both may present with petechiae, purpura, ecchymosis, epistaxis, and other forms of mucocutaneous bleeding [2]. Bone marrow biopsy is essential in establishing the diagnosis. It is a potentially fatal disease, with complications including progression to aplastic anemia, acute myeloid leukemia, myelodysplastic syndrome (MDS), bleeding, and death [2–5]. Fewer than 100 cases of AMT have been described in the literature, primarily as isolated case reports and case series [6]. To evaluate whether acquired AMT (AAMT) occurring in association with chronic lymphocytic leukemia (CLL) had previously been reported, we searched PubMed and Google Scholar from database inception through July 2026 using combinations of the terms “acquired amegakaryocytic thrombocytopenia,” “amegakaryocytic thrombocytopenia,” “AAMT,” “AMT,” “chronic lymphocytic leukemia,” and “CLL.” We were unable to identify prior cases of AAMT occurring in a patient with CLL.

## 2. Case Presentation

An 84‐year‐old man with IgHV‐mutated CLL, diagnosed in 1997, had been managed with observation for many years. Hematologic history was notable for anemia in 2022 and thrombocytopenia in 2023, with no changes to his molecular findings. Computed tomography (CT) of the chest, abdomen, and pelvis in 2022 demonstrated no lymphadenopathy but showed progression of splenomegaly from 13 cm to 16 cm. He received two months of ibrutinib in November 2023 and was restarted in October 2024 due to worsening B symptoms. He presented to the hospital in January 2025 with a 1‐week history of dyspnea on exertion and episodic epistaxis, in addition to a 1‐day history of acute right foot pain. Past medical history included Type 2 diabetes mellitus, hypertension, hyperlipidemia, chronic kidney disease Stage 3, iron deficiency anemia, and gout. He was a former smoker and heavy alcohol user.

On physical exam, there were scattered petechiae throughout the body and erythema on the dorsum of the right foot. Initial laboratory studies found a WBC of 3.2 × 10^3^/μL (decreased from prior level of 16 × 10^3^/μL), hemoglobin (Hgb) of 7 g/dL (decreased from baseline of 10 g/dL), and platelets of less than 1 × 10^3^/μL (decreased from baseline of 50–90 × 10^3^/μL). Additional workup found ESR of 24 mm/hr, CRP of 71 mg/L, LDH of 194 U/L, haptoglobin of 14 g/dL, IgM of 39 mg/dL, negative respiratory viral panel, negative antiphospholipid antibody, anticardiolipin antibody, and lupus anticoagulant. A peripheral blood smear showed anisocytosis, elliptocytosis, occasional schistocytes, decreased WBC, atypical lymphocytes, smudge cells, and no clumping of platelets or presence of giant platelets. CT of the chest, abdomen, and pelvis did not show any acute findings. The patient underwent a bone marrow biopsy for pancytopenia. While awaiting results, the patient received multiple transfusions of RBCs and platelets, with transient improvement in his cell counts. He was treated with empiric antibiotics and was determined to have dry gangrene of the right foot.

**FIGURE 1:**
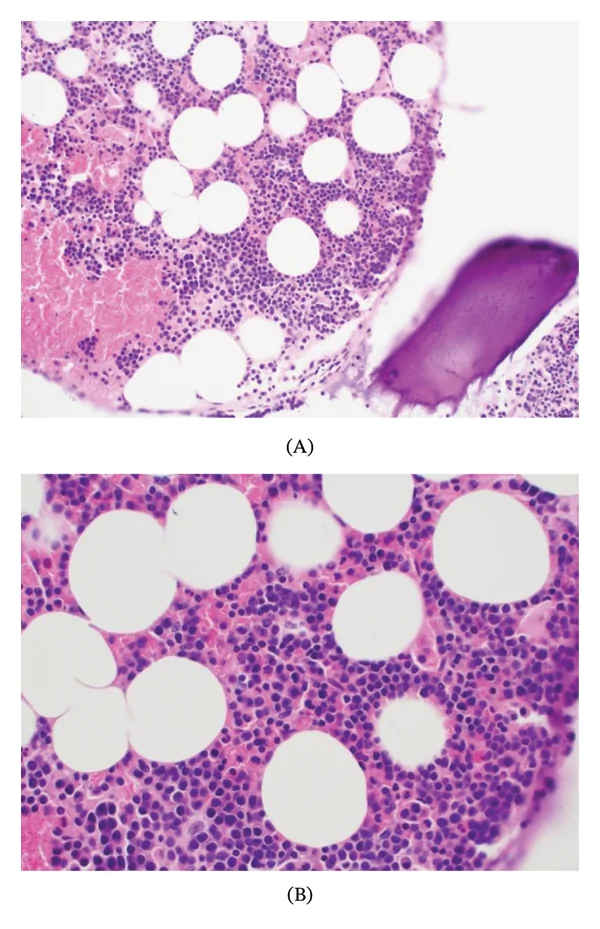
Initial bone marrow biopsy resulted on Hospital day 6. (A) Hematoxylin and eosin stain (× 200 magnification) reveals a relatively hypercellular marrow, showing erythroid hyperplasia, no obvious dysplasia in myeloid or erythroid lineages, and a mild increase in atypical small lymphocytes; no megakaryocytes identified; and no increase in blasts. (B) Higher‐power view (× 400 magnification) confirming the near absence of megakaryocytes.

**FIGURE 2:**
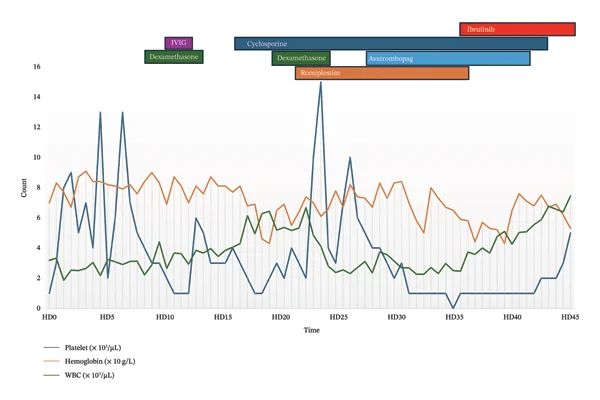
Platelet count, hemoglobin concentration, and white blood cell count are shown throughout the 46‐day hospitalization. Dexamethasone and IVIG were initiated early in the course without sustained platelet improvement, followed by cyclosporine and subsequent addition of romiplostim and avatrombopag for persistent severe thrombocytopenia. Platelet counts remained predominantly below 5 × 10^3^/μL despite overlapping therapies, apart from transient increases, including a rise to 15 × 10^3^/μL after initiation of romiplostim. Ibrutinib was reintroduced later in the hospitalization; a modest late increase in platelet count occurred during overlapping therapy. Color‐coded bars indicate treatment type and duration.

On Day 6 of hospitalization, the biopsy demonstrated a marked decrease in megakaryocytes, no increase in blasts, no obvious dysplasia in myeloid or erythroid lineages, and a mild increase in atypical small lymphocytes, as demonstrated in Figures 1A and B. The patient began a 5‐day trial of high‐dose dexamethasone. Immunoglobulin (IVIG) was added on Hospital day 8; however, his platelet counts further declined. The bone marrow findings and unresponsiveness to steroids and IVIG prompted initiation of cyclosporine after documented platelet response in prior case reports of AMT. Despite the addition of cyclosporine, the patient’s thrombocytopenia remained unresponsive, with platelets persistently averaging 2 to 4 × 10^3^/μL following multiple transfusions. Serial hematologic parameters throughout the hospitalization are summarized in Figure 2 and demonstrate persistent severe thrombocytopenia despite multiple therapeutic interventions. On Hospital day 17, he developed melena and altered mental status; Hgb acutely dropped to 4 g/dL from his inpatient baseline of upper 6–8 s g/dL. The patient was transferred to ICU for severe gastrointestinal bleeding; endoscopy was deferred due to critical thrombocytopenia. Romiplostim was started, and dexamethasone was restarted on Hospital day 18.

A new peripheral blood smear re‐demonstrated absence of platelets. On Hospital day 22, melena subsided, and platelets temporarily improved to 15 × 10^3^/μL, which acutely dropped to 3 × 10^3^/μL the following day. A second thrombopoietin receptor agonist (TPO‐RA), avatrombopag, was given to supplement romiplostim. A trial of rituximab was considered but held off due to concern for adverse effects and limited clinical benefits. On Hospital day 28, his melena returned, and a repeat hemolysis workup was positive for elevated indirect bilirubin and LDH, prompting the discontinuation of all blood products. The patient was transferred out of ICU on Hospital day 33 after stabilization of multiple electrolyte abnormalities.

**FIGURE 3:**
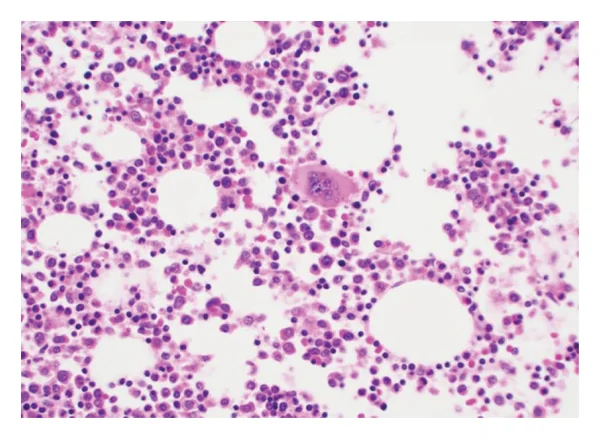
Postmortem bone marrow biopsy: hematoxylin and eosin stain (× 400 magnification) demonstrating persistent marked megakaryocyte depletion with only rare residual megakaryocytes identified, and preserved myeloid and erythroid hematopoiesis. No excess blasts or significant dysplasia are noted.

On Hospital day 45, the patient had an acute episode of severe diffuse abdominal pain with ecchymosis, for which abdominal CT was ordered but could not be performed due to volatile hemodynamics. The patient was found in altered mental status and acute distress the following morning, with a further decline in his hemodynamic stability. He was severely acidotic with a serum bicarbonate level of 11 mmol/L, and his Hgb decreased to 5.3 g/dL. However, platelets steadily improved to 5 × 10^3^/μL from the prior baseline of 1‐2 × 10^3^/μL lasting nearly 2 weeks, as shown in Figure 2. Unfortunately, the patient expired on the same day, 46 days after admission. Autopsy demonstrated evidence of gastrointestinal bleeding and persistent megakaryocyte depletion within the bone marrow, with only rare residual megakaryocytes identified (Figure 3). MYD88‐L265P and CD36 A384fs∗19 frameshift mutations were identified on genomic studies. The patient’s clinical course is summarized in Table 1.

**TABLE 1:** Clinical timeline of CLL–associated AAMT in our patient.

Timeline	Clinical events
1997	Diagnosed with IgHV‐mutated CLL. Managed with observation for over 2 decades
2022	Developed mild anemia without evidence of disease transformation
2023	Developed thrombocytopenia. Received 2 months of ibrutinib, subsequently discontinued
2024	Ibrutinib restarted because of worsening B symptoms
1 week before admission	Progressive dyspnea on exertion and recurrent epistaxis
Hospital day 0	Presented with acute right foot pain and pancytopenia (WBC 3.2 × 10^3^/μL, hemoglobin 7 g/dL, and platelets < 1 × 10^3^/μL). Peripheral smear demonstrated anisocytosis, elliptocytosis, occasional schistocytes, atypical lymphocytes, and smudge cells. CT chest, abdomen, and pelvis showed no acute abnormalities. Platelet and pRBC transfusions produced only transient improvement
Hospital day 6	Bone marrow biopsy demonstrated marked megakaryocyte depletion with preserved erythroid and myeloid maturation, mild atypical lymphoid infiltrate, no excess blasts, and no significant dysplasia, consistent with AAMT. High‐dose dexamethasone initiated
Hospital day 8	IVIG added because of persistent thrombocytopenia
Hospital days 9–16	Cyclosporine initiated following failure of steroids and IVIG. Platelet counts remained 2–4 × 10^3^/μL despite repeated transfusions
Hospital day 17	Developed melena, altered mental status, and acute anemia (hemoglobin 4 g/dL). Transferred to ICU. Endoscopy deferred because of profound thrombocytopenia
Hospital day 18	Romiplostim initiated. High‐dose dexamethasone restarted
Hospital day 22	Platelets transiently increased to 15 × 10^3^/μL before rapidly declining to 3 × 10^3^/μL
Hospital day 23	Avatrombopag added because of inadequate response to romiplostim. Rituximab considered but deferred because of limited anticipated benefit and concern for infectious complications
Hospital day 28	Recurrent melena with laboratory evidence of hemolysis. Blood product transfusions discontinued
Hospital day 33	Stabilized and transferred from ICU to medical floor. Continued intermittent pRBC and platelet transfusions while platelets remained 1‐2 × 10^3^/μL. Ibrutinib reinitiated after exhaustion of other therapeutic options
Hospital day 45	Developed severe diffuse abdominal pain with ecchymosis and progressive hemodynamic instability suspicious for catastrophic intraabdominal hemorrhage
Hospital day 46	Died from hemorrhagic complications despite maximal supportive care. Autopsy demonstrated gastrointestinal hemorrhage and rare residual megakaryocytes within the bone marrow

## 3. Discussion

Given the rarity of AAMT in patients with CLL, distinguishing this entity from more common causes of cytopenia is critical. AMT includes both congenital and acquired forms with distinct underlying mechanisms. Congenital AMT results from mutations in c‐MPL, leading to impaired megakaryocyte development and severe thrombocytopenia that may progress to pancytopenia [2, 7]. AAMT typically presents later in life and is thought to be immune‐mediated [2]. It has been reported in association with autoimmune diseases (e.g., systemic lupus erythematosus, systemic sclerosis, rheumatoid arthritis, and Still’s disease), infections (e.g., Epstein–Barr virus, cytomegalovirus, hepatitis B and C, HIV, and parvovirus B19), hematologic and solid malignancies (e.g., large granular lymphocyte leukemia, thymoma, MDS, and lymphomas), and environmental or iatrogenic exposures (e.g., benzene, alcohol, interferon therapy, radioiodine, and vitamin B12 deficiency) [1, 2, 8]. Notably, autoimmune cytopenias affect up to 10% of CLL patients, most commonly autoimmune hemolytic anemia (AIHA), ITP, and more rarely pure red cell aplasia or neutropenia [9].

Unlike many AAMT cases that mimic ITP, our patient presented with pancytopenia, prompting early marrow evaluation to rule out several additional causes of marrow dysfunction. Aplastic anemia was considered because of the accompanying leukopenia and anemia; however, the bone marrow demonstrated preservation of erythroid and myeloid hematopoiesis rather than global marrow hypocellularity, making established aplastic anemia less likely at the time of presentation. Hypocellular MDS was also considered but was not supported by significant morphologic dysplasia. Drug‐induced marrow suppression remained a potential contributor given recent exposure to ibrutinib and antimicrobial therapy, although selective and profound megakaryocyte depletion would be atypical for generalized medication–related myelosuppression. Viral causes were evaluated, with testing for respiratory pathogens, hepatitis B and C, Epstein–Barr virus, and cytomegalovirus unrevealing. CLL‐related marrow dysfunction was also considered; however, the biopsy demonstrated only mild involvement by atypical small lymphocytes without extensive leukemic replacement. The anemia was additionally compounded by recurrent gastrointestinal bleeding, while infection and treatment effects may have contributed to the leukopenia. Thus, although AAMT best explained the profound thrombocytopenia, the accompanying anemia and leukopenia were likely multifactorial and may have reflected an evolving broader marrow failure process. However, preservation of erythroid and myeloid hematopoiesis on the initial and postmortem bone marrow examination did not support a clear transition to global marrow failure.

Proposed mechanisms of AAMT pathogenesis include immune‐mediated dysregulation, whereby T lymphocytes or autoantibodies target megakaryocyte precursors. Alan G et al. [10] demonstrated that depletion of T lymphocytes restored megakaryocyte colony formation, whereas reintroduction of autologous T lymphocytes reinstated suppression. Autoantibodies against thrombopoietin receptors or megakaryocyte‐associated antigens have also been reported [4]. A stem cell defect impairing megakaryocytic differentiation has also been proposed, linking AAMT to aplastic anemia and other marrow failure syndromes [3–5]. Given the established association between CLL and other autoimmune cytopenias, our case raises the possibility that AAMT represents a rare manifestation of CLL‐associated immune dysregulation; however, a causal relationship cannot be established from a single case. It is plausible that the same mechanisms implicated in CLL‐associated AIHA, ITP, and pure red cell aplasia, including dysregulated B‐cell and T‐cell interactions, cytokine‐mediated immune activation, and loss of peripheral immune tolerance, may have contributed to megakaryocytic suppression in our patient [9]. A modest increase in platelet count was observed following reintroduction of ibrutinib; however, this occurred in the setting of concurrent cyclosporine, steroids, and TPO‐RAs, precluding attribution of the platelet increase to ibrutinib alone.

Treatment of AAMT remains challenging because of its rarity, the absence of standardized guidelines, and variable therapeutic responses. First‐line therapies are typically extrapolated from ITP and include steroids, IVIG, and TPO‐RAs. Other frequently trialed therapies include cyclosporine, rituximab, azathioprine, antithymocyte globulin, vincristine, lithium, danazol, and mycophenolate mofetil [2, 6]. Several prior cases have documented the use of cyclosporine in AAMT patients with variable efficacy [11–14]. By inhibiting calcineurin and T‐cell activation, cyclosporine may suppress immune‐mediated megakaryocyte destruction. Its established role in treating both AAMT and CLL‐associated cytopenias supported its use in our case [12]. TPO‐RAs act by activating gene transcription for megakaryocyte proliferation via binding the c‐MPL receptor. There is evidence that TPO‐RAs can induce hematologic recovery in AAMT, particularly when some residual megakaryocyte precursors are present [4]. Rituximab, an anti‐CD20 monoclonal antibody with immunomodulatory and antilymphoproliferative properties, has shown benefit in AAMT, including those linked to autoimmune cytopenia [15]. While we considered rituximab in our patient, it was withheld due to limited expected benefit and elevated infection risk in the context of clinical decline and transition to palliative care. Ibrutinib, a BTK inhibitor used to induce remission in CLL patients by inhibiting B‐cell receptor signaling [16], was re‐introduced after exhaustion of other therapies. It is known to attenuate autoimmune cytopenias in CLL [17]; however, BTK inhibitor therapy can also be associated with hematologic toxicities, including thrombocytopenia and other cytopenias, further complicating interpretation in this case. Treatment in our patient followed a stepwise approach based on the evolving diagnosis and clinical response. Given the severe thrombocytopenia and bleeding manifestations, IVIG and steroids were initiated empirically for presumed ITP. Following identification of marked megakaryocyte depletion on bone marrow biopsy and lack of response to ITP‐directed therapy, treatment was redirected toward AAMT with cyclosporine and TPO‐RAs. Finally, ibrutinib was reintroduced because of its activity against both CLL and associated autoimmune cytopenias [17]. This case exemplifies the difficulty in balancing immunosuppression for presumed autoimmunity against the need to control the underlying leukemia. Expert consensus generally supports the treatment of CLL‐associated cytopenia first and only escalates to chemoimmunotherapy or targeted therapy in refractory cases [18], consistent with the trajectory in our patient.

In younger patients with fewer comorbidities and greater potential for meaningful recovery, hematopoietic stem cell transplantation remains a final therapeutic consideration. A few case reports have documented successful treatment of AAMT with allogeneic stem cell transplant, including a patient with thymoma‐associated aplastic anemia who progressed from AAMT [19].

Despite an escalation of care, we only observed fleeting rises in our patient’s platelet counts before he succumbed to bleeding complications. This outcome is consistent with prior reports indicating that AAMT carries a high mortality risk, particularly in those with significant comorbidities. While some patients may respond to a combination of immunosuppressive agents, TPO‐RAs, and splenectomy [6], others progress to bone marrow failure or death from hemorrhagic or infectious complications. The need for multiple immunosuppressive treatments can also predispose patients to infections, compounding the risks.

Postmortem genomic analysis identified two mutations: MYD88 L265P and CD36 A384fs∗19. MYD88 is a key adapter protein involved in toll‐like receptor and interleukin‐1 receptor signaling pathways. The MYD88 L265P mutation, commonly associated with Waldenstrom’s macroglobulinemia and several other lymphoproliferative disorders, results in constitutive activation of the NF‐κB pathway, promoting chronic immune activation and lymphoid proliferation [20, 21]. Given the proposed immune‐mediated pathogenesis of AAMT, MYD88‐driven inflammatory signaling may have contributed to the immune dysregulation observed in our patient. Qin et al. reported MYD88 L265P mutation in about 2%–4.4% of Caucasian patients with CLL. In the subgroup with a mutated IgHV gene, as in our patient, this mutation has been associated with inferior survival outcomes [22]. On the other hand, CD36 is a transmembrane scavenger receptor expressed on numerous cells such as platelets, endothelial cells, adipocytes, and hematopoietic progenitor cells. It participates in platelet adhesion and aggregation, thrombospondin‐mediated signaling, and inflammation and immune pathways [23]. Mutations in CD36 have been linked to platelet glycoprotein deficiency and altered platelet function [23]. Although CD36 mutations have not been associated with AAMT, impaired CD36 signaling could theoretically influence platelet biology and immune dysregulation pathways. The significance of the A384fs∗19 mutation is uncertain and warrants further investigation. Although these findings are intriguing, no causal relationship between mutation and AAMT has been established. Their potential relationship to megakaryocyte suppression in this patient is therefore exploratory and hypothesis‐generating.

Several limitations should be acknowledged. As a single case report, this study cannot establish a causal relationship between CLL and AAMT. The patient received multiple therapeutic interventions concurrently or in close succession, limiting our ability to attribute changes in platelet count to any individual treatment, including ibrutinib. Mechanistic studies were not performed to determine whether the MYD88 L265P or CD36 A384fs∗19 alterations contributed to megakaryocyte suppression. Finally, the accompanying anemia and leukopenia were likely multifactorial, and an evolving broader marrow failure process could not be completely excluded despite preservation of nonmegakaryocytic hematopoiesis on the bone marrow biopsy.

## 4. Conclusion

While complications such as AIHA and ITP are well‐documented in CLL [9], their association with AAMT has not been described in the literature to our knowledge. Management of AAMT in the setting of CLL presents a unique clinical challenge, requiring a careful balance between addressing life‐threatening thrombocytopenia with immunosuppressants and/or TPO‐RAs and treating the underlying CLL more aggressively. AAMT remains an underrecognized cause of thrombocytopenia. In patients with CLL and unexplained cytopenias, early hematologic consultation and bone marrow biopsy may facilitate timely diagnosis. Our case highlights the importance of considering AAMT as a diagnostic possibility in CLL patients with unexplained thrombocytopenia and underscores the need for more studies to inform treatment strategies.

## Author Contributions

Christopher C. Chen participated in the conception of the manuscript, interpreted the clinical information, contributed to the literature review, and performed major writing and revision. Anand Shah, Marina Andrawis, Jared Benjamin, Manali Shah, Anthony Fratella‐Calabrese, and Victor T. Chang contributed to the writing and revision of the manuscript. Christopher C. Chen had full access to all of the data in this study and takes complete responsibility for the integrity of the data and the accuracy of the data analysis.

## Funding

No funding was received for this research.

## Disclosure

All authors have read and approved the final version of the manuscript.

## Ethics Statement

As per our institution’s policy, IRB approval was not required due to the nature of this manuscript describing a single patient with no identifiable information.

## Consent

No patient identifiers were used, and the patient was anonymized according to ICMJE guidelines. Verbal consent was obtained.

## Conflicts of Interest

The authors declare no conflicts of interest.

## Supporting Information

Additional supporting information can be found online in the Supporting Information section.

## Supporting information


**Supporting Information** CARE‐checklist‐AAMTCLL.

## Data Availability

The data used to write this manuscript are available from the corresponding author upon reasonable request.
